# A Head-to-Head Comparison of Two Algorithms for Adjusting Mealtime Insulin Doses Based on CGM Trend Arrows in Adult Patients with Type 1 Diabetes: Results from an Exploratory Study

**DOI:** 10.3390/ijerph20053945

**Published:** 2023-02-23

**Authors:** Martina Parise, Sergio Di Molfetta, Roberta Teresa Graziano, Raffaella Fiorentino, Antonio Cutruzzolà, Agostino Gnasso, Concetta Irace

**Affiliations:** 1 Department of Health Science, University Magna Graecia, 88100 Catanzaro, Italy; 2 Section of Internal Medicine, Endocrinology, Andrology, and Metabolic Diseases, Department of Precision and Regenerative Medicine and Ionian Area, University of Bari, 70121 Bari, Italy; 3 Medical School, University Magna Graecia, 88100 Catanzaro, Italy; 4 Diabetes Care Center, University Hospital Mater Domini, 88100 Catanzaro, Italy; 5 Department of Clinical and Experimental Medicine, University Magna Graecia, 88100 Catanzaro, Italy

**Keywords:** type 1 diabetes, continuous glucose monitoring, trend arrows, time in range, glucose variability, bolus, bolus adjustment

## Abstract

Background: Continuous glucose monitoring (CGM) users are encouraged to consider trend arrows before injecting a meal bolus. We evaluated the efficacy and safety of two different algorithms for trend-informed bolus adjustments, the Diabetes Research in Children Network/Juvenile Diabetes Research Foundation (DirectNet/JDRF) and the Ziegler algorithm, in type 1 diabetes. Methods: We conducted a cross-over study of type 1 diabetes patients using Dexcom G6. Participants were randomly assigned to either the DirectNet/JDRF or the Ziegler algorithm for two weeks. After a 7-day wash-out period with no trend-informed bolus adjustments, they crossed to the alternative algorithm. Results: Twenty patients, with an average age of 36 ± 10 years, completed this study. Compared to the baseline and the DirectNet/JDRF algorithm, the Ziegler algorithm was associated with a significantly higher time in range (TIR) and lower time above range and mean glucose. A separate analysis of patients on CSII and MDI revealed that the Ziegler algorithm provides better glucose control and variability than DirectNet/JDRF in CSII-treated patients. The two algorithms were equally effective in increasing TIR in MDI-treated patients. No severe hypoglycemic or hyperglycemic episode occurred during the study. Conclusions: The Ziegler algorithm is safe and may provide better glucose control and variability than the DirectNet/JDRF over a two-week period, especially in patients treated with CSII.

## 1. Introduction

In the last two decades, continuous glucose monitoring (CGM) revolutionized the self-management of diabetes by providing users with near real-time information on their current glucose levels [1,2]. With the ever-increasing accuracy of sensors, more and more systems have been approved for non-adjunctive use by international regulatory agencies, in this way certifying that sensor glucose readings can be safely used for routine diabetes treatment decisions without confirmatory capillary blood testing [3,4].

One of the benefits of CGM is the prediction of future glucose levels with the so-called trend arrows, which indicate both the direction and the rate of change (ROC) of glucose at any given time. However, the meaning of the different arrows may vary depending on the manufacturer [5].

Interpreting trend arrows is a fundamental skill a patient should learn when using CGM. In clinical practice, people with diabetes are told to look at the trend arrows alongside current glucose values before physical activity, driving, bedtime, and before each meal to increase or reduce the calculated meal bolus [6,7]. The changes patients make in mealtime insulin dosage based on trend arrows are largely variable. As illustrated in the survey by Pettus et al., patients would adjust the mealtime dose by an average of 81% and 46% in cases of predicted higher or lower value, respectively [8].

Within the last decade, a number of algorithms have been proposed to determine appropriate dose adjustments based on the trend arrows. However, there is still no consensus due to the lack of robust clinical trials. In two remarkable studies on type 1 diabetes, the Juvenile Diabetes Research Foundation (JDRF) Continuous Glucose Monitoring study and the Diabetes Research in Children Network (DirecNet) Applied Treatment Algorithm study, the ‘10%/20% rule’ for adjusting bolus insulin dose was evaluated for the first time [9,10]. The results of these studies indicated that the use of the ROC might improve the post-prandial glucose level and the quality of life. Later, other authors proposed different algorithms to encourage people with diabetes to handle CGM data daily. According to Scheiner [11] and Pettus and Edelman [12], a defined value ranging from 25 to 100 mg/dL should be added to or subtracted from the current glucose level based on the trend arrow, and a correction bolus should be calculated according to the patient’s insulin sensitivity factor (ISF). Klonoff and Kerr [13] introduced an easy-to-use formula to adjust the meal bolus dose by adding or subtracting the same amount of insulin for all patients, namely 1, 1.5, or 2 insulin units for the ROC of 1–2, 2–3, and >3 mg/dL/min, respectively. Laffel and Aleppo proposed different trend-informed adjustments of bolus doses depending on the individual ISF (<25 mg/dL, 25 to <50 mg/dL, 50 to <75 mg/dL, or >75 mg/dL), with differences between children [14] and adults [15]. Ziegler and colleagues suggested trend-informed bolus adjustments based on both the ISF (the same strategy proposed by Laffel and Aleppo) and pre-meal glucose levels (<70 mg/dL, 70–180 mg/dL, 180–250 mg/dL, or >250 mg/dL) [16]. In 2021, Bruttomesso et al. modified Ziegler’s slide rule by increasing the number of glucose ranges and insulin sensitivity classes [17].

Taking into consideration the role of the ROC as expressed by the trend arrow before calculating the meal bolus, we designed our research to evaluate the short-term efficacy and safety of two different algorithms for bolus adjustments, namely the earlier and simpler DirectNet/JDRF algorithm and the novel (at the time when this study was conducted) and more sophisticated Ziegler algorithm, in a sample of patients with type 1 diabetes using a CGM device.

## 2. Materials and Methods

### 2.1. Study Design

The current research study is an exploratory single-arm, cross-over study. It was approved by the local Ethical Committee, and performed at the diabetes care center of the University Hospital affiliate of the University Magna Graecia, Catanzaro, Italy. Consecutive patients with type 1 diabetes using the Dexcom G6 (Dexcom, Inc., San Diego, CA, USA) CGM system and regularly attending the center were assessed for eligibility. Those who met the inclusion/exclusion criteria (Table 1) were invited to join the study and were enrolled after signing a written informed consent form. Patients’ characteristics and ongoing treatment were retrieved from the electronic medical record.

The study consisted of two 2-week-long intervention phases to evaluate two different algorithms for adjusting the meal bolus based on trend arrows and a 7-day wash-out period with no trend-informed bolus adjustments between the two phases. Algorithm 1 was the simple DirectNet/JDRF, which suggests increasing or reducing the meal bolus, as was previously calculated according to the insulin:carbohydrates ratio (ICR) and ISF, by 10% in the case of a 1–2 mg/dL/min rise or fall in sensor glucose levels and by 20% in the case of a >2 mg/dL/min rise or fall in sensor glucose levels, respectively [10]. Algorithm 2 was the more sophisticated slide rule by Ziegler et al., which suggests changes in the meal bolus according to the trend arrow, pre-meal glucose level (<70 mg/dL; 70–180 mg/dL; 180–250 mg/dL; >250 mg/dL), and individual ISF (<25 mg/dL; 25–<50 mg/dL; 50–<75 mg/dL; >75 mg/dL). When the glucose is changing at a rate >3 mg/dL/min, insulin doses may vary by ±1–3.5 units; when the glucose is changing at a rate of 2–3 mg/dL/min, insulin doses may vary by ±0.5–2.5 units; when the glucose is changing at a rate of 1–2 mg/dL, insulin doses may vary by ±0.5–1.5 units [16]. 

In the two weeks before the study initiation, basal insulin doses, ICR, and ISF were optimized. The sequence of Algorithms 1 and 2 was randomly assigned to each participant. All patients received detailed instructions about the study’s protocol and a scorecard with the proposed adjustments. Patients on multiple daily injections (MDI) therapy were suggested to round the final dose to the lower unit for safety reasons. Participants were also invited not to change their lifestyle throughout the study period and to take three meals without snacks when possible. A follow-up phone call on day 3 of both phases was scheduled to ensure that participants were accurately following the study protocol. 

### 2.2. Outcome Measures

The outcome measures were CGM-derived glucose metrics as recommended by international consensus [16], including time in the 70–180 mg/dL glucose range (TIR), time below range (TBR), time above range (TAR), mean sensor glucose, the standard deviation of mean glucose (SD), coefficient of variation of mean glucose (CV), and the glucose management indicator (GMI). All the glucose metrics mentioned above were downloaded from the Dexcom Clarity platform for healthcare professionals. During this study, all occurrences of severe hypoglycemia, defined as an event requiring the assistance of another person to actively administer carbohydrates, glucagon, or take other corrective actions and severe hyperglycemia, defined as a hyperglycemic event requiring hospitalization, were recorded.

### 2.3. Statistical Analyses

Statistical analyses were performed using SPSS vers.25.0 (IBM, Armonk, NY, USA). The normal distribution of variables was evaluated using the Shapiro–Wilk test. According to the study design, variables were collected and compared at baseline and after Algorithms 1 and 2 (study phases). Patients were analyzed both as a whole and divided according to the type of treatment (MDI or CSII). The ANOVA and the related-sample Friedman’s two-way ANOVA by ranks were used to compare variables collected at baseline and with Algorithms 1 and 2. The Bonferroni post hoc test and Wilcoxon signed rank test were used to compare the glucose metrics between study phases. The sample size was not driven and was arbitrarily chosen to collect adequate information on protocol efficacy and safety.

## 3. Results

Twenty patients with type 1 diabetes, aged 20–61 years, were recruited and completed this study. No severe hypoglycemic or hyperglycemic episodes occurred during the study. The characteristics of the patients enrolled in this study are illustrated in Table 2.

All patients were adherent and wore the sensor more than 70% of the time during each study phase. Glucose metrics collected at baseline and after using the two algorithms are shown in Table 3. TIR, TAR, and mean glucose significantly differed at the three timepoints of the study, while no difference was detected in TBR, SD, CV, and GMI. The post hoc analysis revealed TIR to be significantly higher and TAR and mean glucose to be significantly lower after Algorithm 2 compared to baseline and Algorithm 1.

Three patients (15%) had a TIR > 70% at baseline, whereas five (25%) and twelve (60%) patients had a TIR > 70% after two weeks of using Algorithms 1 and 2, respectively.

The mean insulin dose injected before meals was 15 ± 5 units with Algorithm 2 and 16 ± 6 units with Algorithm 1 (*p* = 0.08).

We then divided the participants according to the therapy regimen, MDI or CSII, and again compared the glucose metrics collected at baseline and the end of the two study phases. The results are displayed in Table 4 and Table 5.

In patients treated with MDI therapy, we found a statistically significant difference in TIR and TBR collected at baseline and after the two study phases. However, TBR was no longer significant (*p* = 0.076) when we excluded one patient with a TBR of 14% at baseline, 7% after Algorithm 1, and 4% after Algorithm 2. Post hoc analyses revealed the TIR to be statistically higher with both algorithms than the baseline.

In patients treated with CSII, TIR, TAR, mean glucose, SD, CV, and GMI were statistically different across the three timepoints of this study. In the post hoc analysis, the same variables differed significantly between Algorithm 2 and the baseline, while Algorithm 1 differed in TIR and TAR when compared to the baseline. Algorithm 1 and Algorithm 2 differed in TIR, TAR, and SD.

## 4. Discussion

Glucose trend arrows add important information for appropriate mealtime insulin dosing in patients with intensive insulin-treated diabetes. However, there is still no consensus on adjusting the scheduled dose according to the upward or downward trend arrows available when using CGM.

In the absence of evidence, healthcare providers recommend increasing or decreasing the amount of insulin injected before a meal by following algorithms proposed by experts or according to self-reported patient experiences. 

To our knowledge, this investigation is the first clinical trial evaluating the efficacy and safety of two different algorithms for adjusting mealtime insulin dose based on trend arrows. Among the algorithms available in the literature when the protocol was submitted to the Ethical Committee, we focused on the simple-to-use algorithm adopted in the DirectNet/JDRF study and the more sophisticated algorithm by Ziegler et al.

In our study, using a structured approach as on-top therapy for adjusting mealtime insulin doses based on trend arrows improved CGM-derived glucose control and variability measures, with the best results obtained by using the Ziegler algorithm in the subgroup of patients treated with CSII. We believe that flexible insulin administration with CSII, allowing for fractions of units to be delivered as a bolus, can magnify the fine-tuned dose adjustments with the Ziegler algorithm. Notably, the results were obtained without the occurrence of severe hypo- and hyperglycemia and without appreciable differences in the total daily bolus insulin dose possibly due to a more appropriate within-day distribution of mealtime doses. However, the DirectNet/JDRF algorithm can be regarded as a valid alternative for increasing TIR in patients on MDI therapy.

Several scientific societies recommend using trend arrows for bolus insulin adjustments for diabetes care [7,14,15,18,19]. Unfortunately, the existence of different algorithms and the lack of guidance for choosing between these methods overcomplicate the clinical scenario.

The major strength of our research is the cross-over study design, which eliminates the influence of environmental factors, eating behaviors, and activity level. Notably, the algorithm was added as on-top therapy; that is, patients had adequate glycemic control at baseline, and basal insulin, ICR, and ISF were all optimized before the study.

The current study has some limitations. Firstly, we only included adult patients with type 1 diabetes; therefore, the applicability of our findings to type 2 patients on intensive insulin treatment or to pediatric patients is unknown. However, Ziegler and colleagues propose dedicated tables for insulin-dependent type 2 diabetes and children/adolescents with type 1 diabetes, possibly resulting in more flexible insulin dosing and the better control of prandial glucose excursions in these groups of patients.

Secondly, we only evaluated the short-term safety and efficacy of the two algorithms. Further research is needed to clarify whether the use of the algorithms may be beneficial in the long term and help patients achieve their desired targets of HbA1c.

The interpretation of trend arrows undoubtedly added a layer of complexity when deciding how much insulin to administer before a meal. Any tool facilitating daily dose calculations may result in the more persistent use of dose adjustment algorithms and the long-term improvement of glucose control. In line with this thought, a visual scorecard reporting discrete amounts of insulin units to either add or subtract to the scheduled insulin bolus, such as the Ziegler et al. algorithm, may be more practical than other methods requiring complex calculations (percent dose increase/decrease or recalculation of meal doses based on predicted glucose levels). The development of CGM-informed bolus calculators (CIBC) with automatic trend-based dose adjustments is a further step towards simplifying daily self-management for patients on intensive insulin-based regimens. The feasibility of such an approach has been recently evaluated in a cohort of twenty-five patients with type 1 diabetes on CSII therapy participating in a two-phase, single-arm, prospective, multicenter study conducted in the U.S. At the end of this study, significantly fewer glucose readings of <70 mg/dL at four hours post bolus were found with the CIBC compared to standard bolus calculation without trend-based adjustment (2.1% ± 2.0% vs. 2.8 ± 2.7, *p* = 0.03), while the percent of readings >180 and 70–180 mg/dL remained the same with no difference in insulin use or the number of boluses given between the two study phases [20]. However, none of the above-mentioned algorithms have been implemented in the automatic bolus calculators currently available on the market, either as a smart phone application or integrated into insulin pumps.

In recent years, the development of closed-loop systems providing glucose-responsive algorithm-driven insulin delivery revolutionized the treatment of type 1 diabetes, with ever-growing evidence highlighting their value in improving TIR, especially in the overnight period, without causing an increased risk of hypoglycemia [21,22,23,24,25,26,27]. Accordingly, international guidelines recommend that these systems be considered either for treating patients with suboptimal glycemia, significant glycemic variability, or impaired hypoglycemia awareness or to allow for permissive hyperglycemia due to the fear of hypoglycemia [2]. In patients treated with these new generation devices, avoiding insulin dose adjustments based on trend arrows is recommended, as algorithms are designed to automatically correct oscillations without external interference [18]. However, access to these new generation devices is still limited. Regional inequalities exist due to a lack of funding, underdeveloped health technology assessment bodies and guidelines, unfamiliarity with novel therapies, and inadequacies in healthcare system capacities [28]. Therefore, the thoughtful use of real-time glucose information, including insulin dose adjustments based on trend arrows, may help maximize glycemic outcomes in the greater proportion of patients using CGM devices [29,30].

## 5. Conclusions

The appropriate interpretation of trend arrows has the potential to maximize glycemic outcomes and improve engagement with diabetes self-management in patients with type 1 diabetes using CGM devices. We conducted a head-to-head comparison between two different algorithms for trend-informed bolus adjustments, and we have shown that the Ziegler algorithm is safe and provides better glucose control and variability than the DirectNet/JDRF, as measured by CGM over two weeks, especially in patients treated with CSII. Further research is needed to clarify whether these benefits are maintained in the long term and may also apply to type 2 patients on intensive insulin treatments or to pediatric patients.

## Figures and Tables

**Table 1:** Inclusion and exclusion criteria.

Inclusion	Exclusion
Age ≥ 18 years	LGS, PLGS, HCL, or AHCL therapy
Diagnosis of type 1 diabetes for at least one year	Pregnancy or lactation
HbA1c < 7.5% measured in the previous three months at the hospital laboratory	Coeliac disease
Stable insulin treatment for at least three months	Current infection
Use of Dexcom G6 system for at least three months	Intense physical activity
Use of ICR and ISF	History of severe hypoglycemia or DKA in the last six monthsUse of steroids or any other drug or condition interfering with glucose control
Naïve to structured trend-informed bolus adjustment	Use of faster-acting insulin analogs

ICR: insulin:carbohydrates ratio; ISF: insulin sensitivity factor; LGS: low glucose suspend; PLGS: predictive low glucose suspend; HCL: hybrid closed loop; AHCL: advanced hybrid closed loop; DKA: diabetic ketoacidosis.

**Table 2:** Characteristics of patients enrolled in the study.

Number of patients (N)	20
Age (years)	36 ± 10
Disease duration (years)	20 ± 8
Gender (Male N)	7
Weight (kg)	70 ± 15
MDI (N)	6
CSII (N)	14
TDD (U)	38.8 ± 11.6
TDD (U/kg body weight)	0.55 ± 0.15
HbA1c (%)	7.3 ± 0.4

Data are expressed as mean ± SD or absolute number. MDI: Multiple daily insulin injections; CSII: continuous subcutaneous insulin infusion; TDD: total daily dose.

**Table 3:** Comparison of glucose metrics collected at baseline and after Algorithm 1 (DirectNet/JDRF) and Algorithm 2 (Ziegler).

Glucose Metric	Baseline	Algorithm 1 (*n* = 20)	Algorithm 2 (*n* = 20)	*p*-Value ANOVA
TIR (%)	59.0 ± 14.3	65.8 ± 12.7	69.0 ± 10.4 *#	0.03
TBR (%)	4.0 ± 4.1	3.7 ± 3.3	3.1 ± 2.7	0.08 ^†^
TAR (%)	37.2 ± 13.9	30.4 ± 12.0	27.7 ± 9.1 *#	0.03
Mean glucose (mg/dL)	162 ± 19	156 ± 16	148 ± 10 *#	0.02
SD (mg/dL)	58 ± 12	54 ± 13	51 ± 11	0.15
CV (%)	36 ± 8	35 ± 6	33 ± 6	0.38
GMI (%)	7.2 ± 0.5	7.0 ± 0.4	6.9 ± 0.3	0.14

TIR: Time in range; TBR: time below range; TAR: time above range; SD: standard deviation; CV: coefficient of variation; GMI: glucose management indicator. Bonferroni post hoc test: * *p* < 0.05 vs. Baseline; # *p* < 0.05 vs. Algorithm 1. ^†^ Related-sample Friedman’s two-way ANOVA.

**Table 4:** Comparison of glucose metrics collected at baseline and after Algorithm 1 (DirectNet/JDRF) and Algorithm 2 (Ziegler) in patients treated with MDI therapy.

Glucose Metric	Baseline	Algorithm 1 (*n* = 6)	Algorithm 2 (*n* = 6)	*p*-Value ANOVA ^†^
TIR (%)	62.3 ± 18.7	69.2 ± 14.6 *	68.8 ± 14.9 *	0.04
TBR (%)	7.3 ± 5.7	5.8 ± 4.1	4.5 ± 3.6	0.04
TAR (%)	32.0 ± 16.4	24.9 ± 12.0	26.5 ± 12.2	0.44
Mean glucose (mg/dL)	155 ± 24	146 ± 13	144 ± 12	0.06
SD (mg/dL)	55 ± 14	53 ± 18	49 ± 17	0.07
CV (%)	35 ± 8	36 ± 10	34 ± 10	0.07
GMI (%)	7.0 ± 0.6	6.8 ± 0.3	6.7 ± 0.3	0.06

MDI: Multiple daily insulin injections; TIR: time in range; TBR: time below range; TAR: time above range; SD: standard deviation; CV: coefficient of variation; GMI: glucose management indicator. ^†^ Related-sample Friedman’s two-way ANOVA. Wilcoxon signed-rank test: * *p* < 0.05 vs. Baseline.

**Table 5:** Comparison of glucose metrics collected at baseline and after Algorithm 1 (DirectNet/JDRF) and Algorithm 2 (Ziegler) in patients treated with CSII therapy.

Glucose Metric	Baseline	Algorithm 1 (*n* = 14)	Algorithm 2 (*n* = 14)	*p*-Value ANOVA ^†^
TIR (%)	57.0 ± 12.6	64.4 ± 12.1 *	69.1 ± 8.5 *#	0.0001
TBR (%)	2.6 ± 2.3	2.8 ± 2.5	2.6 ± 2.2	0.62
TAR (%)	39 ± 12.7	32.7 ± 11.6 *	28.2 ± 7.9 *#	0.0001
Mean glucose (mg/dL)	165 ± 17	160 ± 15	152 ± 8 *	0.02
SD (mg/dL)	59 ± 12	55 ± 10	51 ± 8 *#	0.002
CV (%)	37 ± 8	34 ± 4	33 ± 5 *	0.03
GMI (%)	7.2 ± 0.6	7.1 ± 0.3	6.9 ± 0.2 *	0.02

CSII: Continuous subcutaneous insulin infusion; TIR: time in range; TBR: time below range; TAR: time above range; SD: standard deviation; CV: coefficient of variation; GMI: glucose management indicator. ^†^ Related-sample Friedman’s two-way ANOVA. Wilcoxon signed-rank test: * *p* < 0.05 vs. Baseline; # *p* < 0.05 vs. Algorithm 1.

## Data Availability

The data presented in this study are available upon request from the corresponding author. Most of the data are sensitive and not publicly available.
